# Field validation of different intervention modes for the potential transmission risk of schistosomiasis during post-transmission interruption phase

**DOI:** 10.1371/journal.pntd.0011739

**Published:** 2023-11-06

**Authors:** Jiaxin Feng, Zhaoyu Guo, Peijun Qian, Wenya Wang, Hehua Hu, Xia Zhang, JingBo Xue, Yinlong Li, Chunli Cao, Yuwan Hao, Shizhu Li

**Affiliations:** 1 National Institute of Parasitic Diseases, Chinese Center for Disease Control and Prevention (Chinese Center for Tropical Diseases Research), NHC Key Laboratory of Parasite and Vector Biology, WHO Collaborating Centre for Tropical Diseases, National Center for International Research on Tropical Diseases, Shanghai, People’s Republic of China; 2 Institute for Infectious Disease and Endemic Disease Control, Beijing Center for Disease Prevention and Control, Beijing, China; 3 Jiangling Center for Disease Control and Prevention, Hubei province, People’s Republic of China; 4 School of Global Health, Chinese Center for Tropical Diseases Research, Shanghai Jiao Tong University School of Medicine, Shanghai, People’s Republic of China; Federal University of Agriculture Abeokuta, NIGERIA

## Abstract

**Objective:**

Precision interventions have been proposed in transmission-interrupted areas to further reduce the potential transmission risk of schistosomiasis. This study aimed to evaluate the effects of different interventions modes for potential transmission risk control.

**Methods:**

Three groups of schistosomiasis-endemic villages were selected in Jiangling county, Hubei province. After baseline surveys in 2020, three intervention models were employed in 2021 and 2022. In Model 1, *Oncomelania hupensis* snail control in key settings and an integrated strategy with an emphasis on the infectious sources managing was employed. In Model 2, an integrated health education-led strategy with an emphasis on infectious source management was employed. In Model 3, only the integrated strategy with an emphasis on infectious source management was employed. The effects of the different intervention models were examined with multiple indicators after 2 years of intervention using the entropy-weighted technique for order of preference by similarity to ideal solution (TOPSIS), rank-sum ratio (RSR) and fuzzy combination model of entropy-weighted TOPSIS and RSR.

**Results:**

Entropy-weighted TOPSIS modeling showed that the *C*_*i*_ values of Model 2 were 0.4434, 0.2759, and 0.3069 in the three pilot villages, *C*_*i*_ values were larger, with top comprehensive ranking. The results of the RSR method showed that the RSR values of Model 2 were 0.75, 0.708, and 0.736 in the three pilot villages, with top comprehensive ranking. The results from the fuzzy combination model of entropy-weighted TOPSIS and RSR showed that implementation of Model 2 resulted in the highest comprehensive ranking among the three models in the three pilot villages under C_i_: RSR = 0.1: 0.9, C_i_: RSR = 0.5: 0.5 and C_i_: RSR = 0.9: 0.1.

**Conclusion:**

The integrated health education-led strategy with an emphasis on infectious source management was the optimal model to manage the risk of transmission of schistosomiasis during the post-transmission interruption phase.

## Author summary

Currently, schistosomiasis control has moved into the transmission interruption phase in China. During the transition from the transmission interruption to elimination stages, the national schistosomiasis control program shifts from morbidity control to interventions of schistosomiasis transmission. Following the implementation of intervention models with adaptations to current epidemiological features of schistosomiasis, the shift from conventional comprehensive control to more scientific and precise modern control is an important issue for schistosomiasis control strategies. Therefore, this study selected Jiangling County of Hubei Province, where schistosomiasis was historically hyperendemic and there is a high risk of schistosomiasis transmission, to carry out field empirical research on different transmission risk intervention modes. Results from the RSR, TOPSIS and fuzzy combination model all revealed that the effectiveness of the integrated health education-led strategy with an emphasis on infectious source management was superior to the other two intervention models. Results from this field interventional study showed that the integrated health education-led strategy with an emphasis on infectious source management was the optimal model to manage the risk of transmission of schistosomiasis during the post-transmission interruption phase.

## Background

Schistosomiasis japonica, caused by infection with *Schistosoma japonicum*, is a zoonotic parasitic disease with a large threaten to human health and socioeconomic development in China. Currently, schistosomiasis is mainly prevalent in 12 provinces along and south of the Yangtze River basin, with 100 million people at risk of infection. Following concerted efforts for nearly 70 years, substantial achievements have been made in schistosomiasis control in China [1–3]. Notably, an integrated strategy with an emphasis on the management of the sources of *S*. *japonicum* infections had been implemented for schistosomiasis control since 2004 [4]. As a consequence, transmission control of schistosomiasis was achieved across China in 2015 [5]. By the end of 2021, schistosomiasis elimination was achieved in five provinces, Shanghai, Zhejiang, Fujian, Guangdong and Guangxi, and transmission interruption was achieved in two provinces, Jiangxi and Sichuan. Transmission control was achieved in five provinces, Yunnan, Hubei, Anhui, Jiangxi, and Hunan, with Yunnan and Hubei provinces passing provincial technical assessment of transmission interruption in 2020 [6]. In addition, 449 out of the 452 counties endemic for schistosomiasis (99.34%) achieved transmission interruption or elimination by the end of 2022, and the number of schistosomiasis patients was reduced to 28,565 [7].

Currently, schistosomiasis control has moved into the transmission interruption phase in China. However, schistosomiasis control is difficult to achieve because of the wide host range of wild animals carrying *S*. *japonicum*, the widespread distribution of intermediate host snails [8], low sensitivity of currently available diagnostics and rapid progression towards schistosomiasis control in some endemic foci. Therefore, transmission risk of schistosomiasis will exist in a long period of time, and the endemic status of schistosomiasis may be underestimated in China. Multiple challenges remain to achieve the target for schistosomiasis elimination in all endemic counties proposed in the Healthy China 2030 strategy, and the schistosomiasis control strategy remains to be improved and optimized [9]. During the transition from the transmission interruption to elimination stages, the national schistosomiasis control program shifts from morbidity control to interventions of schistosomiasis transmission. Following the implementation of intervention models with adaptations to current epidemiological features of schistosomiasis, the shift from conventional comprehensive control to more scientific and precise modern control is an important issue for schistosomiasis control strategies [10]. To consolidate current control achievements and further reduce schistosomiasis transmission in marshland and lake regions, different intervention models targeting schistosomiasis transmission were employed in Jiangling county, Hubei province, where schistosomiasis was historically hyperendemic and there is a high risk of schistosomiasis transmission. The aim was to identify the precise intervention model that is suitable for schistosomiasis post-transmission interruption.

## Materials and methods

### Ethics statement

This study was approved by the Ethics Review Committee of the National Institute of Parasitic Diseases, Chinese Center for Disease Control and Prevention (National Center for Tropical Diseases Research) (approval number: 2021019). The written consent was granted from participants or their parents prior to the collection of blood and stool samples, or health education interventions.

### Study area

Jiangling county is a typical marshland and lake region located in central south Hubei province in the middle reaches of the Yangtze River [11,12]. There are multiple ditches, dense vegetation, and frequent rainfall, which is extremely suitable for *Oncomelania hupensis* breeding. Snail habitats measured 2 585.91 hm^2^ in the inner embankments of the county, and all *O*. *hupensis* snails are distributed in ditches. Residents predominantly reside along rivers and frequently contact infested water. Currently, there are 11 townships covering 198 administrative villages in Jiangling county, which are all endemic for schistosomiasis, and the county has been given a high priority for schistosomiasis control.

### Pilot villages

The schistosomiasis-endemic villages were classified into highly, moderately and mildly endemic villages according to an *O*. *hupensis* snail survey and the prevalence of human *S*. *japonicum* infections in Jiangling county from 2015 to 2020. Three highly (Houdang, Yanzhong and Dajunhu), moderately (Xizhuanghu, Dengzhaogang and Shougang) and mildly endemic villages (Xinhe, Chian and Lidian) were randomly sampled as pilot villages.

### Baseline epidemiological surveys of schistosomiasis

Baseline epidemiological surveys of schistosomiasis were performed in pilot villages in 2020, including schistosomiasis examinations, and knowledge, attitude and practice (KAP) questionnaires among permanent residents 6 years of age and older during the slack season. An O. hupensis snail survey was conducted in spring. (1) To investigate the prevalence of human S. japonicum infections, a village group was randomly sampled from each pilot village using a cluster random sampling method. Permanent residents 6 years of age and older were assessed for S. japonicum infections using the indirect hemagglutination assay (IHA). Then, blood samples were collected from seropositive individuals and subjected to the miracidial hatching test with a nylon gauze (three slides for one stool sample) and the Kato-Katz technique. The total number of residents receiving serological tests was no less than 80% of permanent residents. (2) The O. hupensis snail survey was performed in all historical snail habitats and other suspected locations in the pilot villages using systematic sampling combined with environmental sampling for at least 500 frames. Each frame measured 33 cm × 33 cm and had inter-line and inter-frame intervals of both 10 m. S. japonicum infections were identified in O. hupensis snails using microscopy and the loop-mediated isothermal amplification (LAMP) assay. (3) KAP questionnaire: According to the pre-test and literature research, the sample size is calculated to be at least 172 people. Taking the village group as the unit, the method of cluster random sampling was adopted, and at least 100 permanent residents 6 years of age and older were sampled from each village with the questionnaire.

### Field intervention models

**Fig 1:**
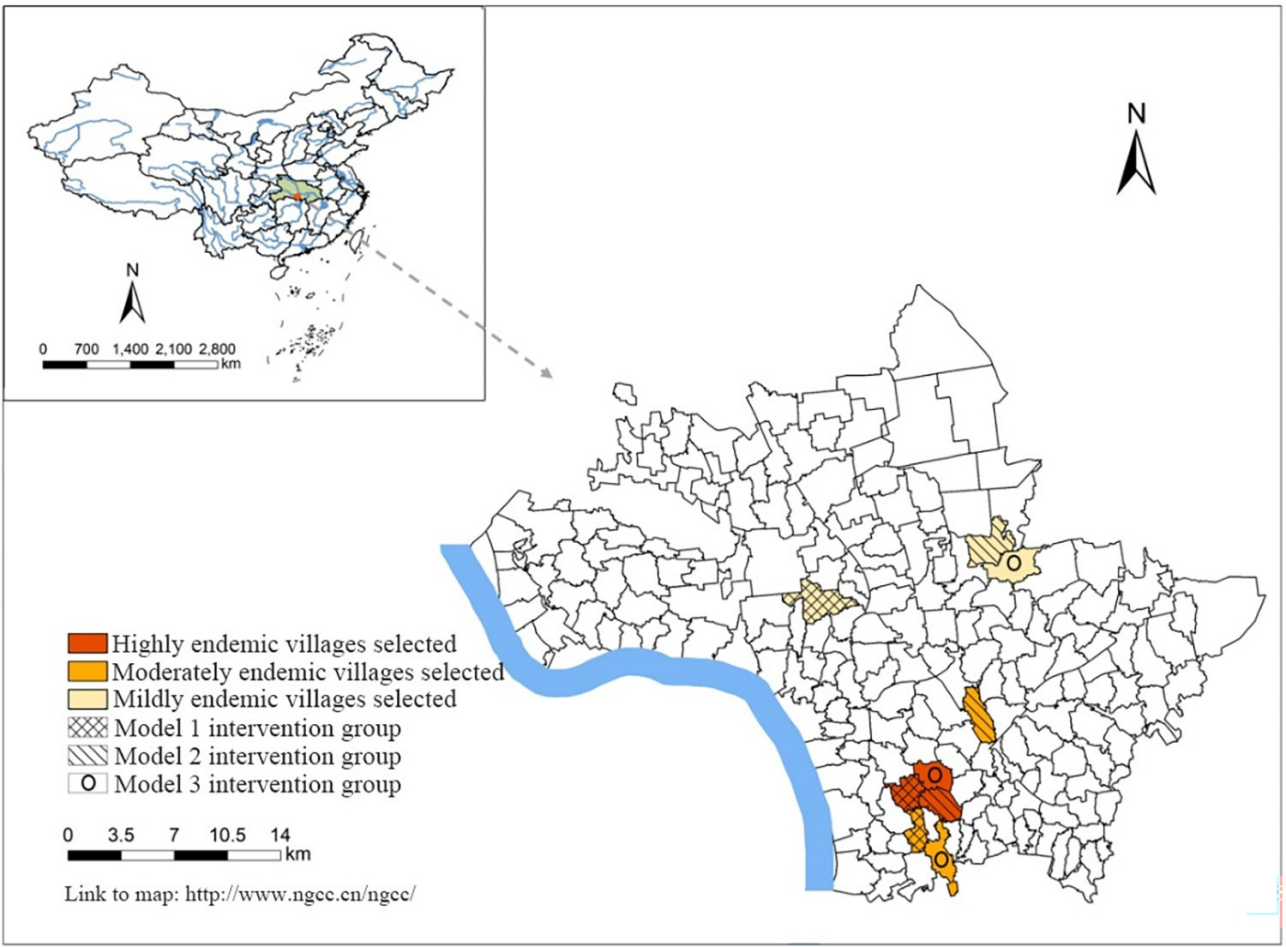
The pilot villages of three models. The base layer of the figure in paper is from http://www.ngcc.cn/ngcc/ with credit to National Geomatics Center of China.

Field demonstration studies for different intervention models were performed from 2021 to 2022. One round of interventions is carried out each year, and a total of two rounds of interventions are carried out, which are divided into the following three modes of intervention. (1) Model 1 was the treatment of *O*. *hupensis* snail-infested settings in key environments plus an integrated strategy with an emphasis on infectious source management. In this model, intensified snail control was performed in key environments in pilot villages (key environments were defined as ditches belonging to independent water systems, which were less than 500 m from residents’ dwellings and had a mean living snail density of more than 2 snails/frame). Chemical treatment combined with engineering snail control in small watersheds was used, while routine chemical treatment with molluscicide spraying was conducted in other settings. According to a randomized approach, Yanzhong, Xizhuanghu and Xinhe were selected as the pilot villages for Model 1 (Fig 1). (2) Model 2 was an integrated health education-led strategy with an emphasis on infectious source management. Detailed interventions included intensified health education for residents living in pilot villages, including face-to-face agricultural lectures and schistosomiasis control lectures, video playing, dissemination of flyers, assignment of electronic alarm devices in snail habitats, and routine posters detailing snail status and schistosomiasis morbidity. According to the randomized approach, Dajunhu, Shougang and Lidian were selected as the pilot villages for Model 2. (3) Model 3 was an integrated strategy with emphasis on infectious source management. Detailed interventions included snail control, posting schistosomiasis control pictures, and posters of snail status and schistosomiasis morbidity. According to the randomized approach, Houdang, Dengzhaogang and Chi’an were selected as the pilot villages for Model 3.

### Evaluation of the effectiveness of intervention models on schistosomiasis transmission

The effectiveness of intervention models for schistosomiasis transmission was evaluated in pilot villages using the same methods described in baseline surveys. A total of eight indicators were employed to determine the effectiveness, including (1) *X*1, the decline in the seroprevalence of human *S*. *japonicum* infections; (2) *X*2, the decline in the number of emerging advanced schistosomiasis patients; (3) *X*3, the decline in the area of snail habitats; (4) *X*4, the decline in living snail density; (5) *X*5, the decline in the occurrence of frames with snails; (6) *X*6, the increase in the rate of correct schistosomiasis prevention knowledge; (7) *X*7, the increase in the rate of correct attitudes towards schistosomiasis prevention; and (8) *X*8, the increase in the rate of correct behaviors towards schistosomiasis prevention.

### Statistical analysis

The entropy method provides weights objectively based on the variations in data of target indicators, and may avoid the impacts of subjective and objective factors on the results [13,14]. The entropy-weighted technique for order of preference by similarity to ideal solution (TOPSIS) is a multi-criteria decision-making method that identifies the superiority and inferiority of the objects according to the difference between the object data and ideal data [15–17]. Following the assignment of weights using the entropy-weight method, parameters were included in the TOPSIS method to rank the effectiveness of different interventions. First, we created a decision matrix with *m* columns and *n* rows (there were nine objects and eight parameters, and therefore, *m* = 9, *n* = 8). In the matrix, *X*_*ij*_ indicates the data of Parameter *j* for Object *i* (*i* = 1, 2, 3, …, *m*; *j* = 1, 2, 3, …, *n*).


$$X=\left[\begin{array}{cccc} x_{11} & x_{12} & \cdots & x_{1n}\\ x_{21} & x_{22} & \cdots & x_{2n}\\ \vdots & \vdots & ... & \vdots \\ x_{m1} & x_{m2} & \cdots & x_{mn} \end{array}\right]$$

The standardized treatment of the matrix and calculation of the proportion were performed using the following formula:

$$Z_{ij}=\frac{x_{ij}-x_{jmin}}{x_{jmax}-x_{jmin}}$$

$$P_{ij}=\frac{z_{ij}}{\sum_{i=1}^{m}z_{ij}}$$

and the entropy value (*e*_*j*_), redundancy (*d*_*j*_) and weight (*w*_*j*_) of each parameter were calculated using the following formula:

$$e_{j}=-\frac{1}{\ln\;m}\sum_{i=1}^{m}P_{ij}\;\ln\;P_{ij}$$

$$d_{j}=1-e_{j}$$

$$w_{j}=\frac{1-e_{j}}{\sum_{i=1}^{n}\left(1-e_{j}\right)}$$

The weighted decision matrix (*Y*) was created using the following formula according to the standardized matrix (*Z*_*ij*_) and weights of each parameter (*w*_*j*_).


$$Y={\left(y_{ij}\right)}_{m\times n}={\left(w_{j}Z_{ij}\right)}_{m\times n}$$

Then, the best (*y*_*j*_^+^) and poorest protocols (*y*_*j*_^−^), namely the highest and lowest effectiveness of intervention models, were evaluated according to the decision matrix (*Y*), and the distances between the parameters of each object and the highest (*D*_*i*_^+^) and lowest effectiveness (*D*_*i*_^−^) using the following formula:

$$y_{j}{}^{+}=\max\{y_{1j},\;y_{2j},\;\ldots ,y_{mj}\},\;y_{j}{}^{-}=\min\{y_{1j},\;y_{2j},\;\ldots ,\;y_{mj}\}$$

$${D_{i}}^{+}=\sqrt{{\sum_{j=1}^{n}\left[w_{j}(y_{ij}-{y_{j}}^{+})\right]}^{2}}$$

$${D_{i}}^{-}=\sqrt{{\sum_{j=1}^{n}\left[w_{j}(y_{ij}-{y_{j}}^{-})\right]}^{2}}$$

The levels of relative proximity from the parameter of each object to the best and poorest protocols (*C*_*i*_) were evaluated using the following formula. A *C*_*i*_ value closer to 1 indicates that the object is approaching the best protocol [18,19].


$$C_{i}=\frac{{D_{i}}^{-}}{{D_{i}}^{+}+{D_{i}}^{+}},\;0\leq C_{i}\leq 1$$

Following the rank transformation of the original matrix (*X*), the dimensionless statistic rank-sum ratio (RSR) was estimated using the following formula, and the effectiveness of the intervention models were ranked according to RSR values.


$${RSR}_{i}=\frac{1}{m\times n}\sum_{j=1}^{m}R_{ij}$$

In this formula, *i* = 1, 2, …, *m*; *j* = 1, 2, …, *n*; and *R*_*ij*_ indicates the rank of the data in the *i* column and *j* row.

Based on the TOPSIS and RSR methods alone, a fuzzy combination model of entropy-weighted TOPSIS and RSR was employed according to the fuzzy set theory in this study. The weight ratio of the *C* value to *RSR* value was set as W1:W2, and the W_1_*C* + W_2_*RSR* was calculated [20,21].

## Results

### Epidemiological characteristics of schistosomiasis before and after intervention

The endemic status of schistosomiasis was determined from nine pilot villages in Jiangling county before and after the implementation of interventions from 2020 to 2022. The seroprevalence of human *S*. *japonicum* infection was 1.35% to 9.5%, and the egg-positive rate was 0% in all pilot villages in 2020 (Table 1). In addition, the occurrence of frames with snails was 0.16% to 0.41%, with no *S*. *japonicum* infection identified in snails (Table 2). Table 3 shows the KAP for schistosomiasis control among residents in pilot villages in 2020, and the proportion of correct behaviors for schistosomiasis control was 43% to 64% at baseline.

**Table 1:** Population survey of pilot areas with different models of transmission risk intervention in 2020.

Intervention model	Pilot villages	No. permanent residents	IHA			Hatching test			Kato-Katz		Emerging advanced schistosomiasis patients	No. patients treated	No. expanded chemotherapy patients
			No. residents receiving serological tests	No. sero-positives	Sero-positives rate(%)	No. residents receiving hatching tests	No. positive hatching tests	Positive hatching tests rate(%)	No. residents receiving Kato-Katz	No. positive Kato-Katz			
Model 1	Yanzhong	415	328	8	2.44	40	0	0.00	40	0	0	3	20
	Xizhuanghu	350	222	18	8.11	32	0	0.00	32	0	0	2	114
	Xinhe	561	444	6	1.35	19	0	0.00	19	0	0	0	153
Model 2	Dajunhu	650	505	48	9.50	111	0	0.00	111	0	0	6	160
	Shougang	410	294	11	3.74	47	0	0.00	47	0	0	2	70
	Lidian	461	353	20	5.67	43	0	0.00	43	0	2	6	68
Model 3	Houdang	455	324	13	4.01	55	0	0.00	55	0	1	3	21
	Dengzhaogang	305	200	15	7.50	34	0	0.00	34	0	2	3	80
	Chian	452	321	21	6.54	60	0	0.00	60	0	0	5	77

IHA: indirect hemagglutination assay

**Table 2:** Snails survey of pilot areas with different models of transmission risk intervention in 2020.

Intervention model	Pilot villages	*O*. *hupensis* snail survey											
		No. survey sites	No. places with snails	Area of survey(hm^2^)	Area of snail habitats (hm2)	Frame of survey	Frame of snail habitats	Occurrence of frames with snails(%)	No. snails	No. living snails	Living snail density	No. detecting snails	No. infected snails
Model 1	Yanzhong	47	40	31.60	11.59	1530	628	0.41	1256	1255	0.82	1255	0
	Xizhuanghu	38	25	17.17	11.77	1283	305	0.24	681	676	0.53	676	0
	Xinhe	44	32	30.00	25.40	1841	551	0.30	1238	1238	0.67	1238	0
Model 2	Dajunhu	50	29	40.78	24.96	3835	1053	0.27	2175	2160	0.56	2160	0
	Shougang	43	41	17.88	17.03	1860	538	0.29	3707	3707	1.99	3707	0
	Lidian	46	45	25.25	24.85	2278	446	0.20	2674	2674	1.17	2674	0
Model 3	Houdang	40	30	38.66	16.86	2115	680	0.32	1338	1331	0.63	1331	0
	Dengzhaogang	32	13	17.93	4.64	1156	190	0.16	461	459	0.40	459	0
	Chian	51	46	24.02	23.42	1663	414	0.25	2963	2963	1.78	2963	0

O. hupensis: Oncomelania hupensis

**Table 3:** Questionnaire survey on schistosomiasis control KAP among residents in pilot areas with different transmission risk intervention models in 2020.

Intervention model	Pilot villages	Questionnaire		KAP		
		No. questionnaires issued	No. valid questionnaires collected	Rate of correct knowledge(%)	Rate of correct attitude(%)	Rate of correct practice(%)
Model 1	Yanzhong	128	126	83.53	86.23	63.03
	Xizhuanghu	119	118	80.13	81.19	55.20
	Xinhe	131	130	78.55	87.07	64.86
Model 2	Dajunhu	157	155	80.04	83.21	56.31
	Shougang	107	106	68.33	75.20	43.55
	Lidian	123	121	87.62	70.36	58.21
Model 3	Houdang	122	120	84.46	88.62	51.30
	Dengzhaogang	123	120	83.20	79.33	61.27
	Chian	125	124	62.88	78.19	44.28

KAP:Knowledge, attitude and practice

From 2020 to 2022, chemical treatment combined with environmental improvements (cutting grasses followed by immersion in molluscicides) was performed in the key ditches of the three pilot villages in Model 1, where a total of 40 settings were given intensified snail control, covering an area of 11.24 hm^2^. Intensified health education interventions were delivered to residents living in the three pilot villages in Model 2, where lectures were given 912 person-times, videos were given 918 person-times, 1 635 flyers were provided and electronic alarm devices were assigned in snail habitats in each pilot village. In addition, chemical treatment over an area of 25.21 hm^2^, nine posters pertaining to schistosomiasis control, and six posters of snail control were provided to the three pilot villages in Model 3.

The seroprevalence of human *S*. *japonicum* infection was 1.34% to 7.67%, and the egg-positive rate was 0% in pilot villages in 2022 (Table 4). In addition, the occurrence of frames with snails was 0.12% to 0.43%, with no *S*. *japonicum* infections detected in snails (Table 5). Table 6 demonstrates the KAP for schistosomiasis control among residents in the pilot villages in 2022, the proportion of correct behaviors for schistosomiasis control was 51% to 83%.

**Table 4:** Population survey of pilot areas with different models of transmission risk intervention in 2022.

Intervention model	Pilot villages	No. permanent residents	IHA			Hatching test			Kato-Katz		Emerging advanced schistosomiasis patients	No. patients treated	No. expanded chemotherapy patients
			No. residents receiving serological tests	No. sero-positives	Sero-positives rate(%)	No. residents receiving hatching tests	No. positive hatching tests	Positive hatching tests rate(%)	No. residents receiving Kato-Katz	No. positive Kato-Katz			
Model 1	Yanzhong	405	302	7	2.32	256	0	0.00	212	0	2	20	80
	Xizhuanghu	363	300	10	3.33	219	0	0.00	135	0	1	10	100
	Xinhe	431	299	4	1.34	245	0	0.00	186	0	0	17	63
Model 2	Dajunhu	551	352	27	7.67	247	0	0.00	195	0	0	27	150
	Shougang	425	307	11	3.58	264	0	0.00	178	0	1	17	58
	Lidian	411	300	14	4.67	258	0	0.00	151	0	0	14	66
Model 3	Houdang	430	302	11	3.64	240	0	0.00	194	0	0	32	100
	Dengzhaogang	389	301	21	6.98	228	0	0.00	145	0	0	21	100
	Chian	443	310	17	5.48	267	0	0.00	110	0	1	17	63

IHA: indirect hemagglutination assay

**Table 5:** Snails survey and control measures of pilot areas with different models of transmission risk intervention in 2022.

Intervention model	Pilot villages	*O*. *hupensis* snail survey												Snails control			
		No. survey sites	No. places with snails	Area of survey(hm^2^)	Area of snail habitats (hm2)	Frame of survey	Frame of snail habitats	Occurrence of frames with snails(%)	No. snails	No. living snails	Living snail density	No. detecting snails	No. infected snails	Environmental improvement	No. of sites for strengthen snails control	Total No. of sites for snails control	Area of snails control(hm^2^)
Model 1	Yanzhong	51	40	57.67	11.59	2286	988	0.43	4146	4146	1.81	4146	0	Shovel sod for soaking	10	39	9.99
	Xizhuanghu	36	25	13.67	11.77	2847	532	0.19	1439	1434	0.50	1434	0	Shovel sod for soaking	24	25	11.77
	Xinhe	43	23	27.33	23.45	3610	1050	0.29	2696	2696	0.75	2696	0	Shovel sod for soaking	6	8	11.28
Model 2	Dajunhu	51	26	44.02	23.54	3760	729	0.19	1595	1583	0.42	1583	0	None	0	26	23.54
	Shougang	43	39	17.88	13.84	1898	466	0.25	2999	2969	1.56	2969	0	None	0	8	3.52
	Lidian	46	42	25.25	21.89	3283	402	0.12	2974	2949	0.90	2949	0	None	0	8	15.88
Model 3	Houdang	32	30	32.19	16.86	2185	747	0.34	3179	3179	1.45	3179	0	None	0	16	8.24
	Dengzhaogang	50	22	27.45	7.81	2991	518	0.17	1259	1255	0.42	1255	0	None	0	22	7.81
	Chian	51	48	24.02	18.62	3265	468	0.14	4152	4126	1.26	4126	0	None	0	5	9.16

O. hupensis: Oncomelania hupensis

**Table 6:** Questionnaire survey and health education measures on schistosomiasis control KAP among residents in pilot areas with different transmission risk intervention models in 2022.

Intervention model	Pilot villages	Questionnaire		KAP			Health education measures							
		No. questionnaires issued	No. valid questionnaires collected	Rate of correct knowledge(%)	Rate of correct attitude(%)	Rate of correct practice(%)	Lecture(person-times)	Video(person-times)	Flyers provided(piece)	Alarm devices assigned	Schistosomiasis control posters(piece)	Snail survey posters(piece)	Snail control posters(piece)	Schistosomiasis situation posters(piece)
Model 1	Yanzhong	120	117	80.42	85.05	63.33	0	0	0	0	2	2	6	7
	Xizhuanghu	80	79	82.25	90.22	58.07	0	0	0	0	2	2	5	8
	Xinhe	118	116	87.71	93.19	60.51	0	0	0	0	2	2	5	7
Model 2	Dajunhu	84	83	91.03	99.04	83.44	300	300	500	1	2	2	5	12
	Shougang	106	105	92.09	85.15	83.21	352	300	576	1	5	2	11	22
	Lidian	107	107	82.24	91.48	74.30	260	318	559	1	5	2	11	22
Model 3	Houdang	100	98	81.11	88.53	58.19	0	0	0	0	2	2	5	7
	Dengzhaogang	100	98	87.35	84.29	51.00	0	0	0	0	2	2	5	8
	Chian	102	101	91.91	82.04	74.03	0	0	0	0	5	2	13	26

KAP:Knowledge, attitude and practice

### Changes in the risk of schistosomiasis transmission before and after intervention

The original data matrix was created based on the indicators used for the assessment of schistosomiasis transmission before and after the implementation of the three intervention models (Table 7). The seroprevalence of human *S*. *japonicum* infection was reduced following the implementation of the three models. The implementation of Model 2 resulted in a decline in the area of snail habitats, density of living snails and occurrence of frames with snails, and a rise in the proportions of correct attitudes and behaviors towards schistosomiasis control.

**Table 7:** Evaluation indicators data of different transmission risk intervention models before and after intervention.

Intervention model	Pilot villages	X1	X2	X3	X4	X5	X6	X7	X8
Model 1	Yanzhong	0.12	-2	0	-0.99	-0.02	-0.03	-0.01	0
	Xizhuanghu	4.78	-1	0	0.03	0.05	0.02	0.09	0.03
	Xinhe	0.01	0	1.95	-0.08	0.01	0.09	0.06	-0.04
Model 2	Dajunhu	1.83	0	1.42	0.14	0.08	0.11	0.16	0.27
	Shougang	0.16	-1	3.19	0.43	0.04	0.24	0.1	0.4
	Lidian	1	2	2.96	0.27	0.08	-0.05	0.21	0.16
Model 3	Houdang	0.37	1	0	-0.82	-0.02	-0.03	0	0.07
	Dengzhaogang	0.52	2	-3.17	-0.02	-0.01	0.04	0.05	-0.1
	Chian	1.06	-1	4.8	0.52	0.11	0.29	0.04	0.3

### Determination of indicators weights using the entropy-weighted method

Table 8 shows the information entropy value, and the redundancy and weight of each parameter used to evaluate the schistosomiasis transmission risk following the implementation of different models. X*1* and X*6* had the highest weights, while X*3* and X*4* had the lowest weights.

**Table 8:** The indicators weight of entropy weight method.

Type	X1	X2	X3	X4	X5	X6	X7	X8
Entropy value	0.7431	0.9316	0.9732	0.9568	0.8515	0.8476	0.8960	0.9101
Redundancy	0.2569	0.0684	0.0268	0.0432	0.1485	0.1524	0.1040	0.0899
Weight of entropy weight	28.86%	7.69%	3.01%	4.85%	16.68%	17.12%	11.68%	10.10%

### Determination of the effectiveness of intervention models using the entropy-weighted TOPSIS method

The weight of each parameter was calculated using the entropy-weighted method, and following multiplication of the weight with the standardized values of parameters, the best and poorest protocols were identified using the TOPSIS method [22]. The best protocol (*y*_*j*_^+^) consisted of the maximum value of each parameter included in the standardized matrix, while the poorest protocol (*y*_*j*_^−^) consisted of the minimum of each parameter included in the standardized matrix.

*y*_*j*_^+^ = (0.2886, 0.0769, 0.0301, 0.0485, 0.1668, 0.1712, 0.1168, 0.1010)

*y*_*j*_^−^ = (0, 0, 0, 0, 0, 0)

The distances from the parameters of each object to the best (*D*_*i*_^+^) and poorest protocol (*D*_*i*_^−^) were estimated, and the levels of the relative proximity from the parameter of each object to the best and poorest protocols (*C*_*i*_) were ranked (Table 9). A higher *C*_*i*_ value indicates that the object was approaching the best protocol. Our findings showed that the *C*_*i*_ values of Model 2 were 0.4434, 0.2759, and 0.3069, respectively, with the highest comprehensive rank among three models, while the *C*_*i*_ values of Model 3 were 0.0993, 0.1477 and 0.4095, respectively, with the lowest comprehensive rank among three models.

**Table 9:** Entropy weight-TOPSIS assessment of the effects of different transmission risk intervention modes.

Intervention model	Pilot villages	*D*_*i*_^+^	*D*_*i*_^−^	*C*_*i*_	Ranking
Model 1	Yanzhong	0.0927	0.0029	0.0303	9
	Xizhuanghu	0.0290	0.0851	0.7461	1
	Xinhe	0.0887	0.0148	0.1428	6
Model 2	Dajunhu	0.0541	0.0431	0.4434	3
	Shougang	0.0814	0.0310	0.2759	5
	Lidian	0.0723	0.0320	0.3069	4
Model 3	Houdang	0.0866	0.0095	0.0993	8
	Dengzhaogang	0.0824	0.0143	0.1477	7
	Chian	0.0656	0.0455	0.4095	2

### Determination of the effectiveness of intervention models using the RSR method

Eight optimal parameters were employed in this study, and the data for each parameter were ranked in a descending order, with mean rank estimated from the same value (Table 7). The RSR value was also estimated and ranked. Table 10 shows the effectiveness of three intervention models of schistosomiasis transmission using the RSR method. The RSR values of Model 2 were 0.75, 0.708, and 0.736 in the three pilot villages with the highest comprehensive rank, while the RSR values of Model 1 were 0.208, 0.556, and 0.451 in the three pilot villages with the lowest comprehensive rank.

**Table 10:** Rank and RSR ranking of evaluation indicators data before and after different transmission risk intervention modes.

Intervention model	Pilot villages	X1	X2	X3	X4	X5	X6	X7	X8	RSR value	RSR ranking
Model 1	Yanzhong	2	1	3	1	1.5	2.5	1	3	0.208	9
	Xizhuanghu	9	3	3	5	6	4	6	4	0.556	5
	Xinhe	1	5.5	6	3	4	6	5	2	0.451	6
Model 2	Dajunhu	8	5.5	5	6	7.5	7	8	7	0.75	2
	Shougang	3	3	8	8	5	8	7	9	0.708	4
	Lidian	7	8.5	7	7	7.5	1	9	6	0.736	3
Model 3	Houdang	4	7	3	2	1.5	2.5	2	5	0.375	8
	Dengzhaogang	5	8.5	1	4	3	5	4	1	0.438	7
	Chian	6	3	9	9	9	9	3	8	0.778	1

RSR: rank-sum ratio

### Determination of the effectiveness of intervention models using the fuzzy combination model of entropy-weighted TOPSIS and RSR

Because of the difference between the entropy-weighted TOPSIS method and the RSR method, there was a variation in the evaluation results. Therefore, a fuzzy combination model of entropy-weighted TOPSIS and RSR may achieve more scientific and reasonable evaluation results (Table 11). Results from the fuzzy combination model of entropy-weighted TOPSIS and RSR showed that the combination ratio of entropy-weighted TOPSIS to RSR at 0.1:0.9 resulted in comprehensive ranks of 2, 4 and 3 for the three Model 2 villages. The combination ratios at 0.5:0.5 and 0.9:0.1 both resulted in comprehensive ranks of 2, 5, and 4 for Model 2, indicating that Model 2 achieved the highest comprehensive ranks in the three pilot villages at all three combination ratios.

**Table 11:** Fuzzy joint evaluation results of entropy weight-TOPSIS method and RSR method.

Intervention model	Pilot villages	Entropy-weighted TOPSIS		RSR		Fuzzy combination model of entropy-weighted TOPSIS and RSR					
		*C*_*i*_	Ranking	RSR	Ranking	Combination model 1^a^	Ranking	Combination model 2^b^	Ranking	Combination model 3^c^	Ranking
Model 1	Yanzhong	0.0303	9	0.208	9	0.1905	9	0.1193	9	0.0481	9
	Xizhuanghu	0.7461	1	0.556	5	0.5746	5	0.6508	1	0.7271	1
	Xinhe	0.1428	6	0.451	6	0.4205	6	0.2971	6	0.1737	7
Model 2	Dajunhu	0.4434	3	0.75	2	0.7193	2	0.5967	2	0.4741	2
	Shougang	0.2759	5	0.708	4	0.6651	4	0.4921	5	0.3192	5
	Lidian	0.3069	4	0.736	3	0.6932	3	0.5215	4	0.3498	4
Model 3	Houdang	0.0993	8	0.375	8	0.3474	8	0.2371	8	0.1268	8
	Dengzhaogang	0.1477	7	0.438	7	0.4085	7	0.2926	7	0.1766	6
	Chian	0.4095	2	0.778	1	0.7409	1	0.5936	3	0.4463	3

a: *C*_*i*_:RSR = 0.1:0.9; b: *C*_*i*_:RSR = 0.5:0.5; c:*C*_*i*_:RSR = 0.9:0.1

## Discussion

Schistosomiasis transmission interruption or elimination was achieved in more than 99% of endemic counties (districts) by the end of 2022, and the current national schistosomiasis control program is moving to the transmission interruption stage [7]. Nevertheless, schistosomiasis is a natural focal disease that is directly associated with the hygienic behaviors of human and livestock. The decline in actions against schistosomiasis may cause the re-emergence of schistosomiasis due to incomplete alterations of epidemiological factors, such as the failure to effectively eradicate the source of *S*. *japonicum* infections and the difficulty in managing the distribution of intermediate host snails [23,24]. Therefore, it is of great practical significance to explore a precise transmission risk intervention model adapted to the epidemic characteristics of schistosomiasis at the stage of transmission interruption for consolidating the existing achievements of schistosomiasis control and continuously promoting the elimination of schistosomiasis.

Comprehensive evaluation is a method of determining the level of priority and inferiority through data processing and the extraction of multiple indicators using mathematical methods, such as modeling [22]. Currently, methods for comprehensive evaluation mainly include TOPSIS, entropy-weighted TOPSIS, the comprehensive index method, the RSR method, and fuzzy evaluation [25,26]. Entropy-weigh TOPSIS and RSR are two commonly used methods for evaluations in multi-target decision-making analyses. Neither method has specific requirements for the data, and have been widely used in multiple health-related fields, including health evaluation, health decision-making, and benefit evaluation [15,27,28]. Entropy-weighted TOPSIS is effective in retaining the original data and allow facilitate the visualization of results, the results can objectively reflect the gap between the evaluation schemes, which is feasible for large sample sizes with multiple evaluation objects and multiple parameters. That approach is also suitable for data with small sample, however, it suffers from problems of impact of the outliers in the measured values [15,27]. The RSR method describes the overall level of objects through the transformation of parameters into nonparametric statistics, which are flexible and convenient. However, the parameters are replaced with ranks during nonparametric transformation, which may cause the loss of original information quantity [29]. A fuzzy combination of entropy-weighted TOPSIS and RSR may supplement the shortcomings of entropy-weighted TOPSIS or RSR alone, and adds to the objectiveness of the results. In addition, a comprehensive analysis of objects with a ratio weight based on the fuzzy set theory may increase the accuracy and sensitivity of the results, which is highly suitable for the comprehensive evaluation of the effectiveness of different intervention models for schistosomiasis transmission.

The indicators that can evaluate the risk of schistosomiasis transmission include several aspects, such as population infection, health literacy, snail distribution, farm cattle infection, field fecal infection, etc. The indicators of human infection include the seroprevalence of human *S*. *japonicum* infections, positive rate of fecal test and the number of newly discovered advanced schistosomiasis, etc. The indicators of health literacy include knowledge accuracy rate, attitude accuracy rate, behavior accuracy rate, behavior compliance rate, etc. The distribution indicators of snails include the area of snail habitats, the area of infected snail habitats, the living snail density, and the occurrence of frames with snails, etc. The infection indicators of cattle include the seroprevalence of cattle *S*. *japonicum* infections and positive rate of fecal test, etc. The indicators of field fecal infection include the positive rate of field fecal, etc. Ding et al. [27] evaluated the prevalence of schistosomiasis in 7 provinces of China using five indicators, including the number of cases and the area of snail habitats detected. We added indicators related to schistosomiasis health literacy in this study to make a more comprehensive assessment of the risk of schistosomiasis transmission. Zhou et al.[30] evaluated the effect of schistosomiasis control by using the rate of human infection, livestock infection and marshland risk indicators. The previous field investigation in this study showed that the infection rate of cattle in Jiangling County was 0, and no field fecal infected with ova of schistosoma was detected. Therefore, this study selected appropriate indicators from the aspects of population infection, snail distribution and health literacy as the outcome indicators for assessing transmission risk, and tried to balance the number of each type of indicators to ensure the scientific results.

However, because these indicators have different dimensions and large numerical differences, a fuzzy combination model of entropy-weighted TOPSIS and RSR was employed for the joint evaluation of the effectiveness of the intervention models [18]. Results from entropy-weighted TOPSIS or RSR alone were the most effective in Model 2, although the effectiveness of different intervention models varied slightly in the pilot villages. Results from the fuzzy combination model of entropy-weighted TOPSIS and RSR showed a significant difference in the effectiveness of the three intervention models. At a combination ratio of entropy-weighted TOPSIS to RSR of 0.1:0.9, the highest effectiveness was seen in Model 2, followed by Model 3 and Model 1. At a ratio of 0.5:0.5, the highest effectiveness was seen in Model 2, followed by Model 1 and Model 2. At a ratio of 0.9:0.1, the highest effectiveness was seen in Model 2, while models 3 and 1 were ranked the same. The results demonstrated that Model 2 achieved the highest comprehensive ranks in the three pilot villages at all three combination ratios. Although there were differences in the estimates and ranks between the fuzzy combination models at the three ratios and other two models, consistent results were generally observed. Results from the RSR, TOPSIS and fuzzy combination model all revealed that Model 2 had the highest rank, suggesting that the effectiveness of the integrated health education-led strategy with an emphasis on infectious source management was superior to the other two intervention models.

In the current study, the results from the three models were highly consistent, and the evaluation results were considered more stable and reasonable. Results from a single-blind, unmatched, cluster-randomized intervention trial with aims to examine the effect of an educational package at rural schools in Linxiang City District, Hunan province, China, showed that the mean score for the knowledge of helminths was 90% higher in the intervention group than in the control group (*P* < 0.001). The incidence of infection with soil-transmitted helminths was 50% lower in the intervention group than in the control group (*P* < 0.001) [31]. Following 17-year health education interventions among female adults in a hyperendemic area of Poyang Lake region, China, a decline was seen in the level of contact with *S*. *japonicum*-infested water and the prevalence of *S*. *japonicum* infection among local female adults [32]. In addition, health education and provisions for safe water have been recommended as major interventions for schistosomiasis control, in addition to chemotherapy [33]. Therefore, health education is effective in reducing the risk of parasitic disease transmission, which is basically consistent with the findings of this study.

Results from this field interventional study showed that the highest effectiveness was achieved using the integrated health education-led strategy with an emphasis on infectious source management in marshland and lake regions where transmission interruption was achieved. This finding may be because the intervention model with improved health education had more rapid effects and more remarkable effectiveness, while the effectiveness of intervention models with an emphasis on intensified snail control or management of source of *S*. *japonicum* infection are more likely to be affected by other factors. Our data showed that a higher snail habitat area, higher snail density, and a higher occurrence of frames with snails was observed in pilot villages in 2022 than in 2020. This may be due to snail diffusion caused by strong rainfalls in 2022, suggesting the necessity to tackle the risk of schistosomiasis transmission in transmission interrupted-regions. Zhang and colleagues comprehensively evaluated the endemic status of schistosomiasis in 10 endemic townships, Dali City, using TOPSIS and RSR methods [34]. Ding et al. [27] examined the prevalence of schistosomiasis in seven endemic provinces of China using TOPSIS and RSR methods. Based on routine evaluations of the endemic status of schistosomiasis, additional interventions were implemented in this study, which can’t only evaluate the endemic status of schistosomiasis in pilot villages but also can examine the effectiveness of different intervention models on schistosomiasis transmission.

Health transmission is one of the important measures for schistosomiasis control. It is a low input, high yield and high benefit measure. It can arouse the whole society to actively pay attention to and support the prevention and control of schistosomiasis, and win more policy support and financial investment. It can also improve people’s compliance with schistosomiasis control efforts. At the same time, it can control the frequency of contact with the infected water of the target population, avoid or reduce the infection of schistosomiasis, which is the basis of primary prevention of schistosomiasis. This study failed to select several specific health education intervention models for research, suggesting that several specific health education intervention models can be selected for comparative study in the future, and the best health education intervention model is proposed.

This study had some limitations. First, the procedures and effectiveness of the interventions of schistosomiasis transmission were affected by multiple factors, including climate. However, such influencing factors were not included in the study. Second, the study evaluated interventions that had been applied for a short period of time, and long-term interventions may be more effective at reducing schistosomiasis transmission. Third, this study did not analyze and compare the three kinds of endemic villages within each intervention mode, and the follow-up analysis will focus on the consistency of intervention measures in different villages and conduct further stratified analysis.

In summary, the results of this study demonstrate the effectiveness of the integrated health education-led intervention model for schistosomiasis control, with an emphasis on infectious source management in the pilot villages using the entropy-weighted TOPSIS and RSR methods. The integrated health education-led model with an emphasis on infectious source management provided precise intervention and management of schistosomiasis in low-prevalence regions. In endemic foci where the transmission of schistosomiasis has been interrupted, the priority of schistosomiasis control model should be immediately changed, with health education prioritized. In addition, an integrated health education-led strategy of reducing schistosomiasis transmission is required and get through the last mile of schistosomiasis elimination, so as to create jointly a community of common health for mankind.

### Ethical considerations

The National Institute of Parasitic Diseases, Chinese Center for Disease Control and Prevention (Chinese Center for Tropical Diseases Research) granted approval for this study (Ethics Approval Number: 2021019).
